# Implementation of digital health in rural populations with chronic musculoskeletal conditions: A scoping review protocol

**DOI:** 10.1371/journal.pone.0291638

**Published:** 2023-12-22

**Authors:** Lara Campos, Daniela Costa, Helena Donato, Baltazar Nunes, Eduardo B. Cruz

**Affiliations:** 1 ESS, Polytechnic Institute of Setúbal, Setúbal, Portugal; 2 National School of Public Health, NOVA University of Lisbon, Lisboa, Portugal; 3 Comprehensive Health Research Center (CHRC), Lisboa, Portugal; 4 Documentation and Scientific Information Service, Centro Hospitalar e Universitário de Coimbra EPE, Coimbra, Portugal; 5 Faculty of Medicine, University of Coimbra, Coimbra, Portugal; 6 Epidemiology Department, Instituto Nacional de Saúde Dr. Ricardo Jorge, Lisboa, Portugal; Iran University of Medical Sciences, ISLAMIC REPUBLIC OF IRAN

## Abstract

Musculoskeletal conditions are a major source of disability worldwide, and its burden have been rising in the last decades. Rural areas, in particular, are associated with higher prevalence of these conditions as well as higher levels of disability, which is likely related to other determinants that affect these communities. Although digital health has been identified as a potential solution to mitigate the impact of these determinants, it is also known that these populations may face barriers that limit the implementation of these interventions. Therefore, the aim of this scoping review is to comprehensively map the evidence regarding the implementation of digital health interventions in rural populations with chronic musculoskeletal conditions. We will include studies published from the year 2000; that report the use of digital interventions that promote prevention, treatment or monitoring of any chronic musculoskeletal condition or chronic pain from musculoskeletal origin, in patients that live in rural areas. This protocol follows the methodological framework for scoping reviews proposed by Arksey and O’Malley, as well as the Joana Briggs Institute (JBI) approach. We will conduct the search on Medline (PubMed), EMBASE, Web of Science and Scopus, as well as grey literature databases. Two independent reviewers will screen titles and abstracts followed by a full-text review to assess the eligibility of the articles. Data extracted will include the identification of the digital interventions used, barriers and enablers identified by the patients or healthcare providers, the patient-level outcomes measured, and the implementation strategies and outcomes reported. By mapping the evidence on the implementation of digital health interventions in rural communities with musculoskeletal conditions, this scoping review will enhance our understanding of their applicability in real-world settings.

## Introduction

Chronic musculoskeletal pain is defined as persistent or recurrent pain that arises as part of a disease process directly affecting bones, joints, muscles, or related soft tissues [1], which result in a highly diverse range of conditions with different etiology, pathophysiology, and impact on physical function [2]. These conditions pose a significant public health concern, as highlighted in the 2019 Global Burden of Disease report, where they ranked sixth in terms of disability-adjusted life-years (DALYs) and held the top position as the primary contributor to years lost to disability worldwide [3].

The disability linked to chronic musculoskeletal diseases increases with age, so, due to the global population aging, this burden has been rising over the years and it´s expected to continue to do so [4]. By virtue of their chronicity and associated disability, they are frequently associated with mental health impairments [5] and with greater risk of subsequent chronic diseases, specially of cardiovascular nature [6]. Also, they are among the largest contributors to the need for rehabilitation services [7].

There is evidence that the burden of these disorders is even more pronounced in rural or remote areas, defined by the Organization for Economic Co-operation and Development (OECD) as communities where the population density is below 150 inhabitants per km^2^ [8]. Although this definition is very diverse across countries and institutions [8], it is broadly recognized that the conceptualization of “rural” is linked to some compositional and contextual variables that are common in these settings, like depopulation, aging, low socioeconomic status, and low education [9], determinants that are linked to higher prevalence and impact of different chronic diseases, including musculoskeletal conditions [10–15].

Furthermore, remote regions frequently face a disadvantage in terms of healthcare provision, as their residents struggle with difficulties in accessing and navigating healthcare services [16, 17], alongside challenges related to transportation difficulties [15]. Additionally, rural populations often exhibit lower levels of health literacy [18], which, in turn, exerts a profound influence on individuals’ capacity to understand and apply health information, and its related to poorer health outcomes and reduced utilization of healthcare services [19].

In an attempt to mitigate this problem, the use of digital technologies has been pointed out as a way to support healthcare delivery, and an effective strategy to overcome care disparities due to geography, supporting consumers to become informed and active participants in their healthcare [4, 20, 21].

Digital health is the concept of using technology, especially internet-based technologies, to diagnose, monitor, treat, and prevent diseases; and include mobile health (mHealth), electronic health records, wearable devices, smartphones applications (Apps), telehealth, telemedicine, artificial intelligence, and robotics [22].

Favorable outcomes associated with digital interventions have been reported in individuals with musculoskeletal conditions [23, 24] and in rural populations with a variety of health disorders [25–27]. Nevertheless, and once again, it is acknowledged that the determinants that characterize rural populations, such as age, socioeconomic level and education, are, on the other hand, linked with difficulties in accessing digital interventions [28] and have been related to lower access and use of digital health [29]. Rural populations have lower levels of digital literacy and lower experience and exposure to technologies [30], which is linked with several barriers that have been associated with the implementation of digital interventions. These barriers encompass various factors, including cultural aspects such as discomfort with the novelty of using technology in rehabilitation and a reliance on traditional manual treatments and face-to-face evaluations [31]. Additionally, technology-related factors, like limited access to internet and digital devices [32], financial constraints such as the cost of accessing internet services or apps [33]; and ethical concerns, surrounding privacy, security, and confidentiality [34], further contribute to these challenges.

Taking this into account, we understand that there is a need to systematically contextualize the use of digital interventions in a very particular setting, the rural populations, understanding not only if they are effective from the clinical point of view but also the characteristics that they should have to be acceptable, positively implementable, and transferable in real world conditions [33, 35].

For this purpose, a scoping review is a particular useful design, since scoping reviews have a broader approach, generally with the aim of mapping and synthesize literature and addressing a broader research question, bringing together literature in disciplines with emerging evidence, as they are suited to addressing questions beyond those related to the effectiveness or experience of an intervention [36].

Therefore, the overarching goal of this scoping review is to comprehensively map the existence evidence regarding the implementation of digital health interventions in rural populations with chronic musculoskeletal conditions. We specifically aim to outline findings related to the type of digital interventions that have been implemented in these populations, their clinical effectiveness, the barriers and enablers that are documented by patients and healthcare providers, and the implementation strategies used and corresponding outcomes. We also intend to identify gaps in the existing evidence about digital health interventions in rural populations with chronic musculoskeletal conditions.

## Materials and methods

This protocol was developed based on the methodological framework for scoping reviews proposed by Arksey and O’Malley [37] and further refined in the approach to the conduct of scoping reviews by the Joanna Briggs Institute (JBI) [36]; and on the Preferred Reporting Items for Systematic Reviews and Meta-Analyses extension for scoping review (PRISMA-ScR) [38]. Additionally, this protocol is registered in the Open Science Framework Registries (https://osf.io/cwsqj).

In line with the aforementioned framework, this review will follow five stages: (1) identifying the research question; (2) identifying relevant studies; (3) study selection; (4) charting the data; (5) collating, summarizing and reporting of the results; and (6) consultation with stakeholders.

### Stage 1: Identifying the research questions

This scoping review aims to comprehensively investigate how digital health interventions have been implemented in rural populations with chronic musculoskeletal conditions or chronic pain from musculoskeletal origin. To fulfill this objective, we formulated our research questions through collaborative discussions with the research team, resulting in a categorization into primary and secondary questions. The primary question that serves as a guide for the review is:

(1) Which digital health interventions–type and purpose–have been developed for individuals with musculoskeletal chronic conditions or chronic pain from musculoskeletal origin and living in rural areas?

Additionally, this scoping review will also focus on the following secondary questions:

(2) What are the patient-level outcomes reported in studies that assess the implementation of digital health intervention in patients with a chronic musculoskeletal condition or chronic pain from musculoskeletal origin and living in rural areas?
(3) What are the enablers and barriers identified in the literature to the implementation of digital health interventions in rural inhabitants with chronic musculoskeletal conditions or chronic pain from musculoskeletal origin?
(4) What implementation strategies have been employed in the delivery of digital health interventions for rural populations with musculoskeletal conditions or chronic pain from musculoskeletal origin, and what were the corresponding implementation outcomes?

### Stage 2: Identifying relevant studies

A three-step strategy for the identification of relevant studies will be used on this review [36].

First, an initial limited search was conducted in April 2023 in PubMed to identify articles on the topic. Second, the text words contained in the titles and abstracts of relevant articles, index terms, Medical Subject Headings (MeSH Terms) and truncation were used to develop a full search strategy (S1 Appendix), that will be performed in PubMed/Medline, EMBASE, Web of Science Core Collection and Scopus electronic databases, with the required adjustments to the characteristics of each one. We will also perform a targeted literature search in grey literature databases (BASE—Bielefeld Academic Search Engine, CORE and MedNar Search Engine).

In the third step, citation tracking will be used to complement the results of database searching. The bibliographic references of all included articles (backward citation tracking), as well as the citing articles of those selected by database searching (forward citation tracking), will be screened.

If relevant, the reviewers will contact the authors of the included studies for further information.

We will initiate contact through email and anticipate a response within a one-month timeframe. In the event of no feedback by the end of this period, a second and final email will be sent.

All the process will be developed with the assistance of a research librarian with an extensive experience in the field.

When submitting the review for publication, a final search will be carried out to check whether potentially relevant literature has been published meanwhile.

### Stage 3: Study selection

The screening process will be guided by the PCC mnemonic (Population, Concept, Context) and will occur in two phases. The first stage will consist in an initial screening of titles and abstracts by two independent reviewers (LC and DC), according to the predetermined eligibility criteria.

Pilot testing to assess reviewer agreement will be performed, randomly selecting 25 titles and abstracts. Screening by both reviewers will start only when is achieved an agreement equal or greater than 75% [39, 40]. Once first phase is completed, the two reviewers will discuss the results and resolving eventual disagreements by consent. If necessary, a third reviewer will be consulted. Once the screening by title and abstract is concluded, it will begin the second stage that will consist in conducting the full-texts review. Once again, disagreements will be resolved by consensus, with a third member of the research team being consulted if agreement is not reached.

All the records will be uploaded to Rayyan, a citation screening software, where any duplicates will be identified and removed.

The results of the search and the study inclusion process will be reported in full in the final scoping review and presented in a Preferred Reporting Items for Systematic Reviews and Meta-Analyses extension for scoping review [PRISMA-ScR] flow diagram [38].

#### Inclusion and exclusion criteria

The inclusion and exclusion criteria will follow the PCP (Population, Concept, Context) format for scoping reviews.

#### Population

We will consider studies that include participants over 18 years, diagnosed with any musculoskeletal chronic disease, like chronic low back pain, chronic neck pain, knee or hip osteoarthritis, rheumatoid arthritis, gout, fibromyalgia, or other rheumatologic condition. We will also contemplate studies that include patients with chronic pain from musculoskeletal origin.

If we come across studies that combine multiple chronic conditions, we will include them only if more than 75% of the participants meet the specified criteria mentioned above.

#### Concept

The key concept of this scoping review is the use of digital interventions in individuals with chronic musculoskeletal conditions or chronic pain from musculoskeletal origin. We will include studies that describe or report the development and/or implementation of any type of digital health interventions—teleconsultation, telemedicine, telerehabilitation, telemonitoring, web-based programs, mobile apps–to promote prevention, treatment, or monitoring of chronic musculoskeletal conditions in patients that live in rural areas. We will consider articles reporting patient-level outcomes, implementation outcomes, and barriers and enablers from the point of view of the patients and/or the caregivers.

We will exclude studies that pertain to the use of digital health for diagnostic purposes, isolated pharmacological prescription, medical imaging, the development of electronic health records, or electronic questionnaire validation.

#### Context

We will exclusively consider studies involving patients with musculoskeletal chronic conditions or chronic pain of musculoskeletal origin residing in rural areas. In cases where studies encompass both rural and urban participants, inclusion will depend on the separate reporting of results.

#### Type of studies

This scoping review will consider empirical studies with qualitative, quantitative, or mixed methods methodologies. Different types of reviews that report the results of empirical studies (systematic, scoping, and narrative reviews) will be consulted to screen the reference lists for potentially relevant studies. Grey literature will include research reports, dissertations and theses, and pilot studies.

We will retrieve all journal articles published from the year 2000, in order to accommodate the wide adoption of digital health following the publication of WHO Vision for digital health [41, 42].

#### Critical appraisal of individual sources of evidence

Scoping reviews typically do not require an evaluation of the methodological quality of the included studies [36]. The decision to undertake a quality appraisal will depend on the quantity and types of sources identified during the search process. If feasible, a comprehensive quality assessment will be performed using the Critical Appraisal Skills Programme (CASP) checklists. These assessments are intended to provide an overview of the overall quality of the evidence without leading to the exclusion of any study uniquely based on its methodological quality.

Two reviewers will independently conduct the critical appraisal. The results will be organized by study type and presented in a tabular form, along with a description of the findings.

To ensure transparent reporting, interrater agreement will be determined using statistical measures such as the Cohen’s κ, and percentage agreement [43, 44].

### Stage 4: Charting the data

After the screening process, the included studies will be uploaded to Covidence, where a predefined data charting template will be created. Prior to starting the data extraction process, two reviewers (LC and DC) will independently conduct a pilot test with five records, aimed to assess the reliability of the reviewers’ extracting data.

The proposed variables are presented in Table 1.

**Table 1:** Variables and definitions for data charting.

VARIABLE	DEFINITIONS
*STUDY CHARACTERISTICS*	
**Reference**	Full reference of the article
**Publication date**	Year the article was published
**Title**	Title of the article
**Country **	The country in which the study is set
*DESCRIPTION OF THE STUDY METHODOLOGY*	
**Objectives**	Objectives of the study
**Study design**	General type of study design. Type of data analyzed: quantitative, qualitative, both
**Study setting**	The location/context where the study takes place
**Population**	Characteristics of the target population of the study.
	Health condition considered in the sample
**Sample size**	Number of participants in the study
**Time points**	The timing of data assessments points
*INTERVENTION*	
**Type of digital intervention**	Type of digital intervention used: teleconsultation, telemedicine, telerehabilitation, telemonitoring, web-based programs, mobile apps,..
**Purpose of digital intervention**	The main clinical goal of the intervention: prevention, treatment, monitoring
**Comparator [if applicable]**	Type of intervention or interventions applied in other treatment groups
*IMPLEMENTATION*	
**Underlying theory/framework**	Identification of the framework or theory that guide the implementation process.
**Barriers and Enablers**	Description of the factors that inhibit or support the implementation of the intervention and the level at which barrier or enabler was analyzed: patient, healthcare provider
**Implementation Strategies**	Type of strategies used to promote/facilitate implementation, based on the Expert Recommendations for Implementing Change (ERIC) Project [45]
**Method**	The type of tools used to evaluate implementation outcomes: survey, interview, administrative data, observation, focus groups, checklist, other
*OUTCOMES*	
**Patient-level outcomes**	Outcomes measured and outcomes measures. Main results in pain intensity, functional disability.
**Implementation-level outcomes**	Outcomes measured, according to Proctor´s Framework [46] (acceptability, adoption, appropriateness, feasibility, penetration, cost, fidelity, sustainment), and the level at which implementation outcome was analyzed: patient, individual healthcare provider, organization [46]; Main results.
*OTHERS*	
**Observations**	Additional observations about the paper

After the pilot test in five studies, differences in charting will be resolved by a third reviewer and the results will be discussed by the research team, to determine the appropriateness of the chart to capture the information needed.

The data extraction form will be updated or refined according to other categories or variables that can emerge as the conduct of the review progresses [36].

Any iterative change or refinement will be clearly detailed and explained in the final scoping review.

### Stage 5: Collating, summarizing and reporting the results

We will conduct the presentation of findings in a tabular form, considering the charted results and their organization by the research questions.

We will use simple descriptive statistics to report quantitative data, such as the number of studies, country of origin, types of studies design, study setting, characteristics of the study population, digital intervention type and comparators.

A descriptive qualitative analysis will be used to describe patient-level outcomes, barriers and enablers reported by the studies and any additional data. Furthermore, principles of framework synthesis, specifically the Expert Recommendations for Implementing Change (ERIC) Project [45] and Proctors Framework [46], will be used to present the data concerning implementation strategies and outcomes, respectively.

A narrative summary of the findings will detail their relation to the research questions and aims of this scoping review.

### Stage 6: Consultation with stakeholders

The consultation with stakeholders is considered an additional source of information, perspectives, meaning, and applicability to the scoping review [37]. Following the recommendations of Levac et al. [47], we established a stakeholder group composed of a patient with a chronic musculoskeletal condition residing in a rural area, a representative from an institutional organization focused on musculoskeletal conditions, a physiotherapist, and a physician with expertise in health interventions in rural settings and rheumatological pathologies.

After completing phase 5, and following ethical approval, we will email stakeholders with a list of preliminary findings, and ask them to answer to a survey that aims to capture their perceptions and experience, helping making sense of study findings. The specific questions of the survey will be decided after the conclusion of the preliminary data analysis, but the main objective is to actively engage respondents with items focused on the research questions.

The data collected through the questionnaires will be analyzed using thematic analysis and reported in the final scoping review.

## Discussion

To our knowledge, this will be the first scoping review that will provide an overview of the current evidence that targets the use and implementation of digital health interventions in rural populations with chronic musculoskeletal conditions or chronic pain from musculoskeletal origin.

The development of the study will be guided by Arksey and O’Malley´s methodological framework for scoping reviews, the Joanna Briggs Institute Methodological Guidelines and the Preferred Reporting Items for Systematic Reviews and Meta-Analysis extension for Scoping Reviews, which will enhance the rigor of the methodology used and the reliability of the findings.

We will undertake a comprehensive search strategy in peer-reviewed databases journals and grey literature databases, that will include a broad range of study designs without language restrictions.

The findings of the proposed review will enable us to situate the implementation of digital interventions in rural populations, encompassing not only their clinical effectiveness but also the necessary attributes for their successful implementation in real-life circumstances.

Obtaining this understanding, we aspire to set the basis for the development of a complex digital intervention in rural populations with a chronic musculoskeletal condition.

## Supporting information

S1 Checklist PRISMA-ScR checklist. (PDF)

S1 Appendix Search strategy. (DOCX)
