# Correction: Implementation of digital health in rural populations with chronic musculoskeletal conditions: A scoping review protocol

**DOI:** 10.1371/journal.pone.0299871

**Published:** 2024-02-27

**Authors:** Lara Campos, Daniela Costa, Helena Donato, Baltazar Nunes, Eduardo B. Cruz

The following information is missing from the funding statement: This project is supported by national funds through CHRC (UIDP/04923/2020).

The updated Funding statement is as follows: This project is supported by national funds through FCT—Fundação para a Ciência e a Tecnologia, I.P., under the PhD grant awarded to LC (PRT/BD/154509/2022) and through CHRC (UIDP/04923/2020).
