# Supplementary material for: Implementation of digital health in rural populations with chronic musculoskeletal conditions: A scoping review protocol
Source: PLoS One. 2023 Dec 22;18(12):e0291638. doi: 10.1371/journal.pone.0291638 (PMC10745161; doi:10.1371/journal.pone.0291638)
Supplement: S1 Appendix — (DOCX) [file pone.0291638.s002.docx]

**APPENDIX 1**

**SEARCH STRATEGY**

| **MEDLINE (Pubmed)** | |
| --- | --- |
| **SEARCH** | **QUERY** |
| #1 | ("Rural Population"[Mesh] OR rural* OR "rural populations" OR "rural areas" OR backcountry OR interior OR "remote areas" OR province OR indigenous) |
| #2 | ("Orthopedics"[Mesh] OR "Musculoskeletal Diseases"[Mesh] OR musculoskeletal OR "Rheumatic Diseases"[Mesh] OR rheumatologic OR rheumatic OR "Back Pain"[Mesh] OR "back pain" OR "Neck Pain"[Mesh] OR "neck pain" OR "Osteoarthritis"[Mesh] OR osteoarthritis OR "Arthritis"[Mesh] OR "Arthritis, Rheumatoid"[Mesh] OR "rheumatoid arthritis" OR "Gout"[Mesh] OR "Gout" OR “Fibromyalgia”[Mesh] OR “musculoskeletal pain” [Mesh] OR “musculoskeletal pain”) |
| #3 | ("digital" OR "Telemedicine"[Mesh] OR "Telerehabilitation"[Mesh] OR "telerehabilitation" OR "eHealth" OR "telehealth" OR "Mobile Applications"[Mesh] OR "app" OR "application” OR "Smartphone"[Mesh] OR "smartphone" OR "mHealth" OR "Internet"[Mesh] OR "internet" OR "remotely" OR "Videoconferencing"[Mesh] OR "videoconference" OR "virtual") |
| #4 | #1 AND #2 AND #3 |
| Limited to from 2000 | |
| **WEB OF SCIENCE CORE COLLECTION** | |
| **#1** | Rural population OR rural OR rural populations OR rural areas OR backcountry OR interior OR remote areas OR province OR indigenous (Topic) |
| **#2** | Orthopedics OR rheumatology OR musculoskeletal diseases OR musculoskeletal OR rheumatic diseases OR rheumatological OR rheumatic OR back pain OR neck pain OR osteoarthritis OR arthritis OR rheumatoid OR gout OR fibromyalgia OR musculoskeletal pain (Topic) |
| **#**3 | Digital OR telemedicine OR telerehabilitation OR eHealth OR mhealt OR telehealth OR mobile applications OR app OR application OR smartphone OR internet OR mobile health OR virtual medicine OR remote consultation OR remotely OR videoconferencing OR videoconference OR virtual OR telecommunications (Topic) |
| **#4** | #1 AND #2 AND #3 AND  **1999**or **1998** or **1994** or **1993** or **1991** (Exclude – Publication Years) |
| **EMBASE and SCOPUS** | |
| #1 | 'rural population':ti,ab,kw OR rural:ti,ab,kw OR 'rural populations':ti,ab,kw OR 'rural areas':ti,ab,kw OR backcountry:ti,ab,kw OR interior:ti,ab,kw OR 'remote areas':ti,ab,kw OR province:ti,ab,kw OR indigenous:ti,ab,kw OR 'indigenous people'/exp OR 'rural population'/exp |
| #2 | orthopedics:ti,ab,kw OR rheumatology:ti,ab,kw OR 'musculoskeletal diseases':ti,ab,kw OR musculoskeletal:ti,ab,kw OR 'rheumatic diseases':ti,ab,kw OR rheumatologic:ti,ab,kw OR rheumatic:ti,ab,kw OR 'back pain':ti,ab,kw OR 'neck pain':ti,ab,kw OR osteoarthritis:ti,ab,kw OR arthritis:ti,ab,kw OR rheumatoid:ti,ab,kw OR gout:ti,ab,kw OR fibromyalgia:ti,ab,kw OR 'musculoskeletal pain':ti,ab,kw OR 'musculoskeletal pain'/exp OR 'fibromyalgia'/exp OR 'musculoskeletal disease'/exp OR 'arthritis'/exp OR 'osteoarthritis'/exp OR 'rheumatic disease'/exp |
| #3 | digital:ti,ab,kw OR telemedicine:ti,ab,kw OR telerehabilitation:ti,ab,kw OR ehealth:ti,ab,kw OR telehealth:ti,ab,kw OR 'mobile applications':ti,ab,kw OR app:ti,ab,kw OR application:ti,ab,kw OR smartphone:ti,ab,kw OR mhealth:ti,ab,kw OR internet:ti,ab,kw OR 'mobile health':ti,ab,kw OR 'virtual medicine':ti,ab,kw OR 'remote consultation':ti,ab,kw OR remotely:ti,ab,kw OR videoconferencing:ti,ab,kw OR videoconference:ti,ab,kw OR virtual:ti,ab,kw OR telecommunications:ti,ab,kw OR 'telecommunication'/exp OR 'videoconferencing'/exp OR 'teleconsultation'/exp OR 'telehealth'/exp OR 'telemedicine'/exp OR 'smartphone'/exp OR 'mobile health application'/exp OR 'mhealth'/exp OR 'telerehabilitation'/exp OR 'digital health'/exp |
| #4 | #1 AND #2 AND #3 AND [2000-2023]/py |
